# Comparative Studies of Interactions between Fluorodihydroquinazolin Derivatives and Human Serum Albumin with Fluorescence Spectroscopy

**DOI:** 10.3390/molecules21101373

**Published:** 2016-10-14

**Authors:** Yi Wang, Meiqing Zhu, Feng Liu, Xiangwei Wu, Dandan Pan, Jia Liu, Shisuo Fan, Zhen Wang, Jun Tang, Risong Na, Qing X. Li, Rimao Hua, Shangzhong Liu

**Affiliations:** 1 Department of Science of Pesticides, School of Resources and Environment, Anhui Agricultural University, No. 130 Changjiang West Road, Hefei 230036, China; wangyi@ahau.edu.cn (Y.W.); zhumeiqing@ahau.edu.cn (M.Z.); wxw@ahau.edu.cn (X.W.); dandanpan@ahau.edu.cn (D.P.); ljia198346@163.com (J.L.); fanshisuo@ahau.edu.cn (S.F.); zwang@ahau.edu.cn (Z.W.); tangjun@ahau.edu.cn (J.T.); risongna@163.com (R.N.); 2 Department of Applied Chemistry, China Agricultural University, No. 2 Yuanmingyuan West Road, Beijing 100193, China; caulf0527@163.com; 3 Collaborative Innovation Center of Henan Grain Crops, National Key Laboratory of Wheat and Maize Crop Science, College of Plant Protection, Henan Agricultural University, Wenhua Road No. 95, Zhengzhou 450002, China; 4 Department of Molecular Biosciences and Bioengineering, University of Hawaii, 1955 East-West Road, Honolulu, HI 96822, USA; qingl@hawaii.edu

**Keywords:** synthesis, fluorine, fluorescence quenching, human serum albumin, FDQL

## Abstract

In the present study, 3-(fluorobenzylideneamino)-6-chloro-1-(3,3-dimethylbutanoyl)-phenyl-2,3-dihydroquinazolin-4(1*H*)-one (FDQL) derivatives have been designed and synthesized to study the interaction between fluorine substituted dihydroquinazoline derivatives with human serum albumin (HSA) using fluorescence, circular dichroism and Fourier transform infrared spectroscopy. The results indicated that the FDQL could bind to HSA, induce conformation and the secondary structure changes of HSA, and quench the intrinsic fluorescence of HSA through a static quenching mechanism. The thermodynamic parameters, Δ*H*, Δ*S*, and Δ*G*, calculated at different temperatures, revealed that the binding was through spontaneous and hydrophobic forces and thus played major roles in the association. Based on the number of binding sites, it was considered that one molecule of FDQL could bind to a single site of HSA. Site marker competition experiments indicated that the reactive site of HSA to FDQL mainly located in site II (subdomain IIIA). The substitution by fluorine in the benzene ring could increase the interactions between FDQL and HSA to some extent in the proper temperature range through hydrophobic effect, and the substitution at meta-position enhanced the affinity greater than that at para- and ortho-positions.

## 1. Introduction

Human serum albumin (HSA) is the most abundant plasma protein in the circulatory system of the human body and contains 585 amino acids. HSA plays a key function in physiological and pharmacological processes. For example, HSA can transport several endogenous and exogenous compounds, like fatty acids, nutrients, steroids, certain metal ions, hormones and drugs, and also significantly affect their biological activity in pharmacology through altering their pharmacokinetic properties [1,2,3,4,5,6,7]. Binding of drugs to plasma proteins may strongly influence their distribution and elimination [8], for instance, many drug candidates were rendered ineffective due to their unusually high binding affinity to HSA [9,10,11,12,13,14]. Therefore, investigation of the binding interaction between drugs and HSA is of importance in pharmacology and pharmacodynamics.

Heterocyclic structures play a pivotal role in many pharmaceutical, agrochemical, and veterinary products, like quinazolines. An increasing number of quinazoline derivatives have been reported to possess antibacterial [15], anticancer [16], antitubercular [17], anti-HIV [18], antifungal [19], analgesic [20], anti-inflammatory [21], anticonvulsant [22], antiparkinson [23], anthelmintic [24], anti-histaminic [25], and antihypertensive activities [26]. Much attention has been focused on their structures and bioactivity. Little information is, however, available on the interactions of quinazolines with HSA. Fluorine substituents have often been introduced in developing pharmaceutical products and agrochemicals to improve the biological activity and metabolism. However, it is hard to estimate whether the compounds are effective with introducing a fluorine substituent by molecule designing. Especially because there is no information about the impact of fluorine substituent position on the interactions between quinazoline derivatives and HSA.

**Scheme 1:**
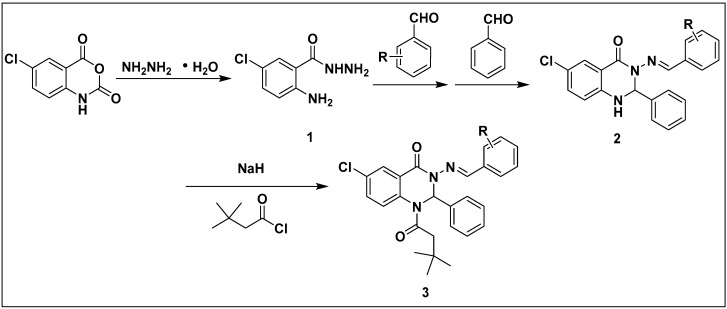
Synthetic route of 6-Chloro-3-fluorobenzylideneamino-2-phenyl-2,3-dihydroquinazolin-4(1*H*)-one (FDQL). R = H (**3a**), *p*-F (**3b**), *m*-F (**3c**), *o*-F (**3d**). *p*, para; *m*, meta; *o*, ortho; and F, fluorine.

We have previously reported the impact of halogen substituents on the interactions between quinazoline derivatives and HSA [27]. In the present study, new 6-chloro-1-(3,3-dimethylbutanoyl)-3-(fluorobenzylideneamino)-phenyl-2,3-dihydroquinazolin-4(1*H*)-one (FDQL) derivatives (**3b**–**d**) (Scheme 1) were designed and synthesized, and their binding interactions to HSA at three temperatures were investigated via fluorescence spectroscopy. The obtained information about the molecular interactions, including quenching mechanism, binding mode, binding sites, binding constants, thermodynamic parameters, and conformation of HSA may offer a better understanding of its biological action in vivo. The influence of the position of fluorine substituent on FDQL-HSA interactions was studied under simulated physiological conditions using 3-(benzylideneamino)-6-chloro-1-(3,3-dimethyl-butanoyl)-2-phenyl-2,3-dihydroquinazolin-4(1*H*)-one as a reference compound. The results provide a quantitative understanding of fluorine substitution effects on FDQL-HSA interactions to some extent, which could be useful for further design of potential biologically active quinazolinone derivatives.

## 2. Results and Discussion

### 2.1. Synthesis

In the previous work, the introduction of *t*-butylacetyl moieties on quinazolines improved their binding to HSA to some extent through hydrophobic action [27]. Thus, based on **3a** that was previously reported, we designed and synthesized compounds **3b**–**d** to study the impact of fluorine substituents on the interactions of HSA and quinazolines. These new compounds have been characterized with NMR and HRMS spectra (see Supplementary Materials).

### 2.2. Fluorescence Quenching Mechanism

**Figure 1:**
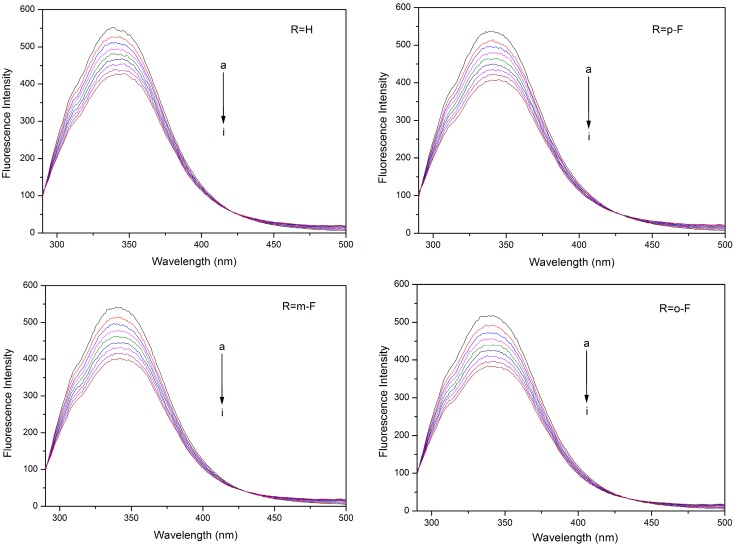
Fluorescence quenching spectra of HSA at different concentrations of FDQL at 298 K; λ_ex_ = 280 nm; C(HSA) = 1.0 × 10^−6^ M; C(FDQL)/(×10^−6^ M), (a–i) 0.000, 1.333, 2.667, 4.000, 5.333, 6.667, 8.000, 9.333, and 10.667 respectively.

The effect of FDQL on the fluorescence emission spectra of HSA was first tested with a fluorescence spectrophotometer, and obtained data were plotted in Figure 1. This figure clearly displays that the fluorescence intensity of HSA consistently decreased in the presence of increasing concentration of FDQL due to fluorescence quenching by FDQL, and a complex formation between FDQL and HSA.

In general, the fluorescence of HSA could be dynamically or statically quenched by an organic compound. As to which one works herein, some variable tests about temperature, viscosity, and lifetime measure were employed and the Stern-Volmer Equation (1) [28] were used to analyze the data for determining the mechanism of quenching fluorescence of HSA by FDQL.

*F*_0_/*F* = 1 + *K*_SV_ [Q] = 1 + *K*qτ_0_ [Q] (1)

where *F*_0_ and *F* are the fluorescence intensities of HSA without and with the quencher, respectively, *K*_SV_ is the linear Stern-Volmer quenching constant, *K*q is the bimolecular quenching constant, [Q] is the concentration of the quencher, τ_0_ is the average lifetime of the biomolecule in the absence of the quencher (τ_0_ = 10^−8^ s^−1^) [29]. The Stern-Volmer plot curves were linear with high values, and the calculated *K*_SV_ and *K*q at the corresponding temperatures were listed in Table 1.

It is known that linear Stern-Volmer plots indicate one type of quenching mechanism as predominant, either static or dynamic [30]. Moreover, in Table 1, the values of *K*q at different temperatures were much higher than the limiting diffusion rate constant of biomolecules (*k*_d_ ≈ 2.0 × 10^10^ M^−1^·s^−1^) [31,32,33,34], which revealed static quenching via forming a complex.

**Figure 2:**
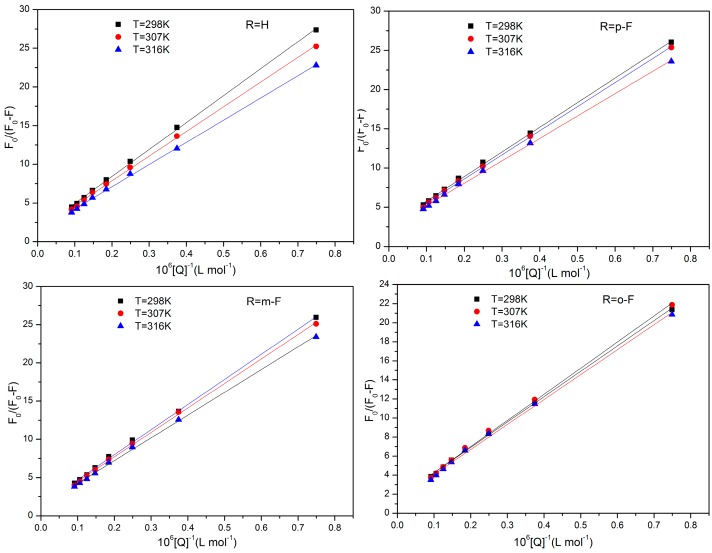
Modified Stern-Volmer plots of FDQL-HSA systems.

For a complex formation process, the affinity constant *K*_a_ of the binding between FDQL and HSA was analyzed by modified Stern-Volmer Equation (2) [35,36,37]:

*F*_0_*/*(*F*_0_*− F*) *= f*_a_*^−^*^1^·*K*_a_^−1^*·*[Q]^−1^*+ f*_a_^−1^ (2)

where *f*_a_ represents the fraction of accessible fluorescence and *K*_a_ is the effective quenching constant. *F*_0_*/*(*F*_0_ *− F*) is linear with the reciprocal value of the quencher concentration [Q], and the slope equals to the value of *f*_a_^−1^·*K*_a_^−1^. According to the modified Stern-Volmer Equation, data were calculated to obtain the linear plots at different temperatures shown in Figure 2. The corresponding values of *K*_a_ in Table 2 showed that the affinity constants all increased after substitution by fluorine atom on the benzene ring.

### 2.3. Binding Sites and Identification of Binding Sites on HSA

For the static quenching process, the number of binding sites can be obtained on the double-logarithmic equation [38,39,40]

lg[(*F*_0_ − *F*)/*F*] = lg*K*_b_ + *n*lg[Q] (3)

where *F*_0_ and *F* are the fluorescence intensities without and with the ligand, and *K*_b_ and n are the binding constant and the number of binding sites, respectively. According to Equation (3), the number of binding sites *n* can be determined on the slope of a straight line produced by the plots of lg[(*F*_0_ − *F*)/*F*] versus log[Q]. The data shown in Table 3 demonstrated a good linear relationship and *n* approximates to 1, indicating that only one site in HSA is reactive to FDQL.

HSA is a globular protein composed of three homologous α-helical domains (I–III), and each domain contains two subdomains (A and B). The principal regions of ligand binding sites on HSA locate in hydrophobic cavities in subdomains IIA and IIIA [41]. Sjöholm et al. [42] reported that phenylbutazone (PB), flufenamic acid (FA), and digitoxin (Dig) bind subdomain IIA (site I), subdomain IIIA (site II), and site III, respectively, which were used in the present study as the site markers in competitive experiments to identify the binding sites of FDQL on HSA. The fluorescence quenching data in the presence of site markers were analyzed with the modified Stern-Volmer Equation, and the values of binding constants listed in Table 4 showed a remarkable decrease after the addition of FA, but relatively small changes after the addition of PB and Dig. Therefore, FDQL would be mainly bound to HSA in site II (subdomain IIIA).

### 2.4. Thermodynamic Parameters and Binding Modes

The interaction forces between ligands and biomolecules include probably electrostatic interactions, multiple hydrogen bonds, van der Waals force, hydrophobic and steric contacts, and so on [43]. Generally, the signs and magnitudes of the thermodynamic parameters enthalpy change (Δ*H*) and entropy change (Δ*S*) can account for the main forces involved in the binding process.

If Δ*H* does not vary significantly in the temperature range studied, both Δ*H* and Δ*S* can be evaluated from the Van′t Hoff equation:

ln*K*_a_ = −Δ*H*/*RT* + Δ*S*/R (4)

where *K*_a_ is analogous to the associative binding constants at the corresponding temperature and R is the gas constant.

**Figure 3:**
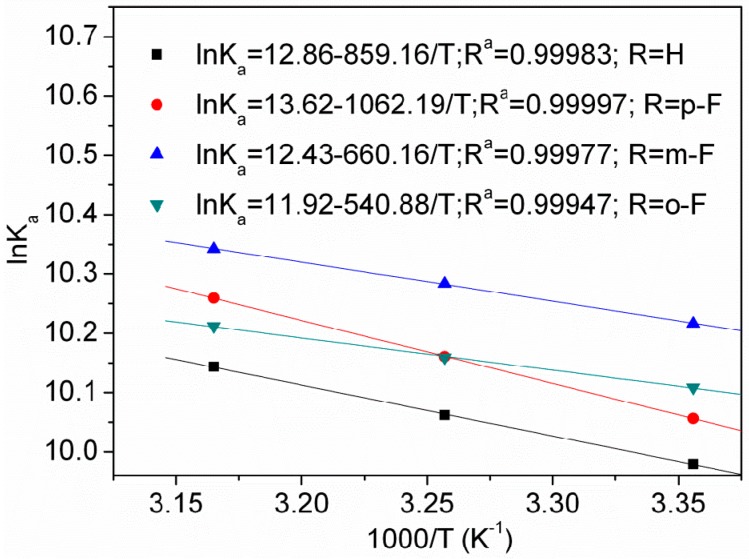
Van′t Hoff plots for FDQL-HSA systems.

To elucidate the interaction between FDQL and HSA, the thermodynamic parameters were calculated from the Van′t Hoff plots (Figure 3). Δ*H* was calculated from the slope of the Van′t Hoff relationship. The free energy change (Δ*G*) was then estimated from the following equation:

Δ*G* = Δ*H* − *T*Δ*S* (5)

The negative signs for free energy (Δ*G*) of the FDQL-HSA systems indicated that the interaction processes were spontaneous (Table 2). The signs for Δ*H* and Δ*S* of the binding reaction were both found to be positive, which indicated that the binding was mainly entropy-derived and the enthalpy was unfavorable for it, according Ross and Subramanian [44]. Thus, the hydrophobic forces played a major role in the binding process of FDQL to HSA.

With use of 3-(benzylideneamino)-6-chloro-1-(3,3-dimethylbutanoyl)-2-phenyl-2,3-dihydro quinazolin-4(1*H*)-one as a reference compound, the changes of Δ*H* and Δ*S* (ΔΔ*H* and ΔΔ*S*, respectively) were compared and studied after incorporating different fluorine substituent positions in the benzene ring. The signs of ΔΔ*H* and ΔΔ*S* listed in Table 5 showed that the binding affinity was enhanced by hydrophobic interaction after incorporating para-fluoro atoms, but by van der Waals force after incorporating meta-fluoro and ortho-fluoro atoms upon on the thermodynamic law summarized by Ross and Subramanian.

In addition, the data at the corresponding temperature in Table 6 displayed the Δ*G* changes (ΔΔ*G*) of interactions of FDQL-HSA after incorporating fluorine atom on the benzene ring. The values showed that the ΔΔ*G* changed only slightly as the temperature changed, and the negative sign of ΔΔ*G* indicated that incorporation of substitution fluorine atom increased the binding affinity in the FDQL-HSA systems. Furthermore, it was found that the meta-fluoro substitution enhanced the affinity more greatly than that of para-fluoro and ortho-fluoro substitution.

### 2.5. HSA Conformational Change by CD and FT-IR Measurements

**Figure 4:**
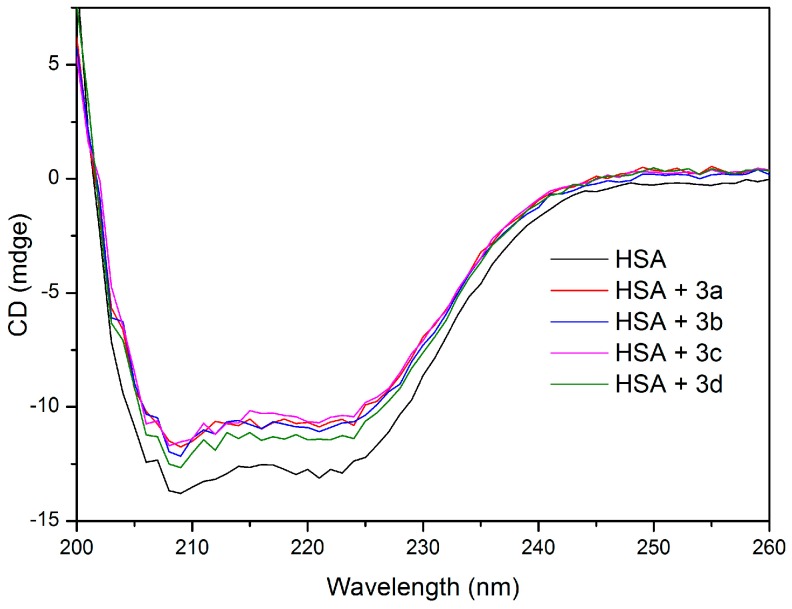
Far-UV CD spectra of the FDQL–HSA system: C_HSA_ = 1 μM and 1 μM HSA + 2 μM FDQL.

Currently, CD spectroscopy is a sensitive technique to provide some information about the secondary structure of proteins. In order to explore any molecular conformation changes of HSA occurring in the binding process, the Far-UV CD measurements for HSA in the absence and presence of FDQL were carried out in the range of 200–260 nm. The CD spectra of HSA and FDQL-HSA complex were shown in Figure 4 which exhibited two negative bands in the UV region at 208 nm and 222 nm, showing characteristic of α-helix structure units of protein [45]. The intensities of the negative bands increased with the addition of FDQL without a change in the position and shape of peak. The calculated results of the fractions of α-Helix, β-sheet, β-Turn, and Random Coil structures were listed in Table 7. These results indicated that the structure of HSA after addition of FDQLs is still predominantly α-helix. Overall, FDQL-HSA complex forms in the solution and the binding process can induce some secondary structure change of HSA.

**Figure 5:**
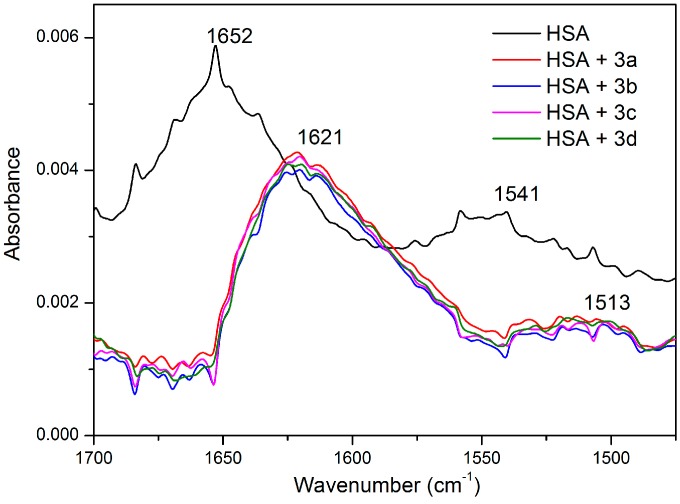
FTIR spectra of free HSA (10 μM) and the FDQL-HSA complex ([FDQL] = 20 μM).

FT-IR spectroscopy was performed to confirm the CD results, and structural changes of HSA after binding with FDQL were observed with FT-IR (Figure 5). According to the literature [46], the FT-IR spectra for HSA monitored over a range of 1700–1500 cm^−1^ reveal the presence of two bands, the amide I band (1700–1600 cm^−1^, mainly C=O stretch) and the amide II band (1600–1500 cm^−1^, a C-N stretch coupled with N-H bending mode), which are related to the secondary structure of the protein. After addition of FDQLs to HSA, the peak position of amide I was shifted from 1652 cm^−1^ to 1621 cm^−1^, and amide II was moved from 1541 cm^−1^ to 1513 cm^−1^. In addition, the intensity of the amide I and amide II band decreased and the peak shape changed, indicating that FDQLs interacted with the C=O and C-N groups in the protein structural subunits. These observations jointly support that FDQLs could induce conformational changes in HSA during the binding process, which agreed with the results of CD experiments.

## 3. Experimental Section

### 3.1. General Methods and Materials

Melting points were measured with a Fisher-Johns melting point apparatus (Cole-Parmer Co., Shanghai, China) without correction. Nuclear magnetic resonance (NMR) spectra were recorded with a 400-MHz spectrometer (Bruker, Billerica, MA, USA) and 600-MHz spectrometer (Agilent Technologies, Inc., Santa Clara, CA, USA) using tetramethylsilane (TMS) as an internal standard. IR spectra were recorded on a Bruker Tensor 27 (Bruker Optics GmbH, Ettlingen, Germany) in KBr pellets in the range 4000–400 cm^−1^. Mass spectra were recorded using an HPLC-1100/TOF MS high resolution mass spectrometer (Agilent Technologies). All fluorescence spectra were measured on a Cary Eclipse fluorescence spectrophotometer (Agilent Technologies) equipped with a thermostat bath. Quartz cuvettes with a 1-cm path length and 3-mL volume were used for all measurements. All pH measurements were completed with a PHS-25 digital pH meter (Shanghai REX Instrument Factory, Shanghai, China). HSA (≥99.9, fatty-acid free), purchased from Sigma-Aldrich (St. Louis, MO, USA), was used without further purification. PB, FA, and Dig were of analytical grade, and purchased from the National Institute for Control of Pharmaceutical and Bioproducts (Beijing, China), and the stock solutions were prepared in absolute ethanol. All other commercial reagents were used as obtained from Sigma-Aldrich, Alfa Aesar (Ward Hill, MA, USA), and J&K (Beijing, China). Flash column chromatography with silica gel was used to purify the crude products.

### 3.2. Fluorescence Titration Experiments

All HSA solutions were prepared in buffer solution (0.1 M Tris base and 0.1 M NaCl at pH 7.4), and the HSA stock solutions were kept in the dark at 4 °C. The solution (3.0 mL) containing 1.0 × 10^−6^ M HSA was titrated by successive additions of 8.0 × 10^−4^ M ethanol stock solution of FDQL (the final concentration is 1.333–10.667 × 10^−6^ M). Titrations were done manually by using trace syringes, and the fluorescence intensity was measured (excitation at 280 nm and emission at 337 nm). All experiments were conducted at three temperatures (298, 307, and 316 K).

### 3.3. Site Marker Competitive Experiments

Binding location studies between FDQL and HSA in the presence of three site makers (phenylbutazone, flufenamic acid, digitoxin) were measured according to the fluorescence titration methods. The concentration of HSA and site makers were all stabilized at 1.0 × 10^−6^ M. The solution of FDQL was then gradually added to the phenylbutazone-HSA, flufenamic acid-HSA, or digitoxin-HSA mixtures, and the fluorescence intensity was recorded (λ_ex_ 280 nm and λ_em_ 337 nm).

### 3.4. Circular Dichroism Spectra Studies

The CD measurements were performed on a Jasco-810 spectropolarimeter (Jasco, Japan) at 298 K with a thermostatically controlled cell holder attached to a NeslabRTE-110 water bath with an accuracy of ±0.1 °C. The instrument was sufficiently purged with 99.9% dry nitrogen gas and calibrated with d-10-camphorsulfonicacid before starting the apparatus. Each spectrum was performed with the use of a quartz cuvette of 0.1 cm path length and taken at wavelengths between 200 and 260 nm with 1 nm step resolution and the average of five successive scans recorded at a speed of 50 nm·min^−1^ and response time of 1 s. All observed CD spectra were baselines subtracted for buffer and the secondary structure was computed using the Jasco standard spectra analysis software package.

### 3.5. Fourier Transform Infrared (FT-IR) Measurements

FT-IR measurements were carried out at room temperature on a Thermo Scientific Nicolet iS50 FTIR spectrometer (Thermo, Tewksbury, MA, USA) equipped with a germanium attenuated total reflection (ATR) accessory. All spectra were taken via 32 scans with a resolution of 4 cm^−1^. The FT-IR spectra of HSA in the absence and presence of FDQL were first collected in the range of 500 cm^−1^–4000 cm^−1^ and the absorbance of the buffer solution (Tris-HCl buffer solution at pH 7.40) was then subtracted.

### 3.6. Synthesis of (un)Substituted Phenyl-2,3-dihydroquinazolin-4(1H)-one Derivatives FDQL ***3a**–**d***

Compound **1** and compounds **2a**–**d** were prepared according to the reported methods [47,48]. Their properties were as follows:

*2-Amino-5-chlorobenzohydrazide* (**1**): white crystals, yield 85%; m.p. 139.6–140.5 °C; ^1^H-NMR (400 MHz, DMSO-*d*_6_) δ 9.60 (s, 1H), 7.48 (d, *J* = 2.5 Hz, 1H), 7.16 (dd, *J* = 8.8, 2.5 Hz, 1H), 6.74 (d, *J* = 8.8 Hz, 1H), 6.46 (s, 2H), 4.42 (s, 2H).

*3-(Benzylideneamino)-6-chloro-2-phenyl-2,3-dihydroquinazolin-4(1H)-one* (**2a**): light yellow solid, yield 75.3%; m.p. 193.5–194.5 °C; ^1^H-NMR (400 MHz, Chloroform-*d*) δ 9.16 (s, 1H), 7.86 (d, *J* = 2.4 Hz, 1H), 7.61–7.52 (m, 2H), 7.40–7.34 (m, 2H), 7.33–7.22 (m, 6H), 7.21–7.17 (m, 1H), 6.58 (d, *J* = 8.6 Hz, 1H), 6.21 (s, 1H), 4.82 (s, 1H).

*6-Chloro-3-(2-fluorobenzylideneamino)-2-phenyl-2,3-dihydroquinazolin-4(1H)-one* (**2b**): light yellow crystal, yield 63.7%; m.p. 193.3–194.1 °C; ^1^H-NMR (400 MHz, Chloroform-*d*) δ 9.25 (d, *J* = 1.2 Hz, 1H), 7.85 (d, *J* = 2.5 Hz, 1H), 7.39–7.32 (m, 2H), 7.29 (d, *J* = 2.1 Hz, 1H), 7.28–7.23 (m, 5H), 7.19 (d, *J* = 1.8 Hz, 1H), 7.02–6.96 (m, 1H), 6.59 (d, *J* = 8.6 Hz, 1H), 6.20 (s, 1H), 4.82 (s, 1H).

*6-Chloro-3-(3-fluorobenzylideneamino)-2-phenyl-2,3-dihydroquinazolin-4(1H)-one* (**2c**): light yellow crystal, yield 60.5%; m.p. 204.3–206.1 °C; ^1^H-NMR (400 MHz, Chloroform-*d*) δ 9.13 (s, 1H), 7.91–7.80 (m, 1H), 7.61–7.49 (m, 2H), 7.42–7.30 (m, 2H), 7.31–7.23 (m, 3H), 7.21–7.16 (m, 1H), 7.00–6.92 (m, 2H), 6.64–6.53 (m, 1H), 6.23–6.15 (m, 1H), 4.88–4.77 (m, 1H).

*6-Chloro-3-(4-fluorobenzylideneamino)-2-phenyl-2,3-dihydroquinazolin-4(1H)-one* (**2d**): white crystal, yield 65.3%; m.p. 200.1–202.0 °C; ^1^H-NMR (400 MHz, Chloroform-*d*) δ 9.32 (s, 1H), 7.90–7.85 (m, 1H), 7.73 (td, *J* = 7.5, 1.8 Hz, 1H), 7.38–7.31 (m, 2H), 7.28–7.24 (m, 4H), 7.20–7.16 (m, 2H), 7.05–6.95 (m, 2H), 6.58 (d, *J* = 8.6 Hz, 1H), 6.23 (d, *J* = 1.9 Hz, 1H), 4.86 (s, 1H).

For the preparation of compounds **3a**–**d**, the corresponding compound **2** (3 mmol) in anhydrous tetrahydrofuran (30 mL) was cooled to 0 °C with an ice bath, and sodium hydride (3.6 mmol) was added. The mixture was stirred for 0.5 h at 0 °C followed by another 1.0 h at room temperature. *tert*-Butylacetyl chloride (3.6 mmol) in anhydrous tetrahydrofuran (5 mL) was added slowly at 0 °C in 0.5 h, and then the mixture was stirred again at room temperature overnight. The solvent was removed under vacuum, and the residue was then purified by flash chromatography using hexane and ethyl acetate (*v*/*v* = 6:1) as the eluent to obtain the title compounds.

*3-(Benzylideneamino)-6-chloro-1-(3,3-dimethylbutanoyl)-2-phenyl-2,3-dihydroquinazolin-4(1*H*)-one* (**3a**): white solid, yield 49.5%; m.p. 149.5–150.8 °C; ^1^H-NMR (600 MHz, DMSO-*d*_6_) δ 9.17 (s, 1H), 7.81 (m, 4H), 7.61 (s, 2H), 7.52–7.41 (m, 3H), 7.33–7.13 (m, 5H), 2.67 (s, 2H), 0.95 (s, 9H); ^13^C-NMR (151 MHz, DMSO-*d*_6_) δ 171.56, 158.59, 152.50, 136.82, 136.54, 134.42, 133.39, 131.45, 130.95, 129.34, 129.27, 129.02, 128.16, 128.01, 127.57, 126.33, 125.90, 44.73, 32.14, 29.86; HRMS: *m/z* calc for C_27_H_26_ClN_3_O_2_ [M + H]^+^ 460.1786, found 460.1776

*6-Chloro-1-(3,3-dimethylbutanoyl)-3-(2-fluorobenzylideneamino)-2-phenyl-2,3-dihydroquinazolin-4(1H)-one* (**3b**): white solid, yield 53.3%; m.p. 172.2–173.0 °C; ^1^H-NMR (600 MHz, DMSO-*d*_6_) δ 9.17 (s, 1H), 7.96–7.66 (m, 4H), 7.61 (s, 2H), 7.52–7.38 (m, 1H), 7.37–7.11 (m, 6H), 2.79–2.57 (m, 2H), 0.95 (s, 9H); ^13^C-NMR (151 MHz, DMSO-*d*_6_) δ 171.56, 158.55, 136.83, 130.50, 130.44, 129.36, 129.28, 129.03, 128.17, 126.33, 125.85, 116.55, 116.40, 44.72, 39.76, 32.14, 29.86; IR (v·cm^−1^): 3677.1, 2958.4, 1891.5, 1670.9, 1603.8, 1511.2, 1371.9, 1315.9, 1233.3, 1164.6, 839.6, 768.9, 755.1, 700.0, 594.9, 540.1; HRMS: *m/z* calc for C_27_H_25_ClFN_3_O_2_ [M + H]^+^ 478.1692, found 478.1680.

*6-Chloro-1-(3,3-dimethylbutanoyl)-3-(3-fluorobenzylideneamino)-2-phenyl-2,3-dihydroquinazolin-4(1H)-one* (**3c**): white solid, yield 50.9%; m.p. 114.2–116.0 °C; ^1^H-NMR (600 MHz, DMSO-*d*_6_) δ 9.20 (s, 1H), 7.81 (s, 2H), 7.71–7.55 (m, 4H), 7.54–7.44 (m, 1H), 7.38–7.10 (m, 6H), 2.67 (m, 2H), 0.95 (s, 9H); ^13^C-NMR (151 MHz, DMSO-*d*_6_) δ 171.56, 158.69, 150.38, 137.05, 136.87, 133.51, 131.46, 131.41, 129.32, 129.28, 129.04, 128.15, 128.05, 127.61, 126.31, 125.77, 124.54, 118.20, 118.06, 114.09, 113.94, 47.77, 44.73, 32.13, 29.85; IR (v·cm^−1^): 3430.0, 2957.5, 1693.5, 1668.1, 1479.5, 1421.4, 1359.6, 1278.3, 1232.0, 1146.0, 755.9, 704.9, 542.2; HRMS: *m/z* calc for C_27_H_25_ClFN_3_O_2_ [M + H]^+^ 478.1692, found 478.1677.

*6-Chloro-1-(3,3-dimethylbutanoyl)-3-(4-fluorobenzylideneamino)-2-phenyl-2,3-dihydroquinazolin-4(1H)-one* (**3d**): white solid, yield 56.3%; m.p. 148.0–149.1 °C; ^1^H-NMR (600 MHz, DMSO-*d*_6_) δ 9.56 (s, 1H), 7.99–7.86 (m, 1H), 7.84–7.66 (m, 2H), 7.62 (s, 2H), 7.54 (m, 1H), 7.47 (m, 1H), 7.34–7.15 (m, 6H), 2.67 (s, 2H), 0.96 (s, 9H); ^13^C-NMR (151 MHz, DMSO-*d*_6_) δ 171.57, 162.43, 160.76, 159.08, 158.58, 145.92, 136.78, 134.42, 133.54, 131.46, 129.35, 129.31, 129.27, 129.07, 128.16, 128.02, 127.59, 127.12, 126.33, 126.27, 125.77, 125.51, 125.49, 122.05, 116.71, 116.57, 44.73, 32.10, 29.86, 29.82; IR (v·cm^−1^): 3423.2, 2962.9, 1670.1, 1481.4, 1431.8, 1359.8, 1125.8, 830.8, 755.8, 596.9; HRMS: *m/z* calc for C_27_H_25_ClFN_3_O_2_ [M + H]^+^ 478.1692, found 478.1682.

## 4. Concluding Remarks

In summary, interactions of four FDQLs with HSA have been studied with fluorescence spectroscopy, CD, and FT-IR experiments. The experimental results indicated that the FDQL could bind to HSA in the site II (subdomain IIIA), induce conformation and the secondary structure changes of HSA and quench the intrinsic fluorescence of HSA through a static quenching mechanism. The quenching of HSA fluorescence takes place with 1:1 complex formation between FDQL and HSA. In the complex formation between HSA and FDQL, hydrophobic forces play a significant role. The studies of the FDQL-HSA interactions showed that substitution of a fluorine atom on the benzene ring could enhance the binding affinity through the steric and hydrophobic effects; specifically, meta-fluoro substitution enhanced the binding affinity most greatly by hydrophobic interaction. This study has provided some valuable information for further research into the rational design of this series of compounds. As the functions of the same substituent in aromatic compounds are relatively consistent and stable, the obtained results about varying fluorine substitution positions are hopefully valuable for guiding molecular design and modification of benzene rings.

## Figures and Tables

**Table 1:** Stern-Volmer quenching constants for interactions of FDQL with HSA at different temperatures.

Compound	T (K)	*K*_SV_ (×10^4^ M^−1^)	*K*q (×10^12^ M^−1^·s^−1^)	*R* ^a^	*SD* ^b^
R = H	298	2.77	2.77	0.99916	0.00605
	307	2.98	2.98	0.99898	0.00677
	316	3.23	3.23	0.99919	0.00513
R = *p*-F	298	3.11	3.11	0.99931	0.00451
	307	3.25	3.25	0.99933	0.00466
	316	3.52	3.52	0.99946	0.00453
R = *m*-F	298	3.24	3.24	0.99941	0.00436
	307	3.44	3.44	0.99889	0.00633
	316	3.63	3.63	0.99924	0.00553
R = *o*-F	298	3.30	3.30	0.99973	0.00299
	307	3.36	3.36	0.99942	0.00449
	316	3.40	3.40	0.99907	0.00573

^a^ *R* is the correlation coefficient. ^b^ *SD* is the standard deviation for the *K*_SV_ values.

**Table 2:** Thermodynamic parameters of FDQL-HSA binding systems at different temperatures.

Compound	T (K)	*K*_a_ (×10^4^ M^−1^)	*R* ^a^	Δ*H* (kJ·mol^−1^)	Δ*G* (kJ·mol^−1^)	Δ*S* (J·mol^−1^·K^−1^)
R = H	298	2.16	0.99949	7.14	−24.72	106.92
	307	2.34	0.99969		−25.68	
	316	2.54	0.99963		−26.65	
R = *p*-F	298	2.33	0.99945	8.83	−24.92	113.24
	307	2.59	0.99967		−25.93	
	316	2.85	0.99967		−26.95	
R = *m*-F	298	2.73	0.99979	5.49	−25.31	103.36
	307	2.92	0.99960		−26.24	
	316	3.10	0.99967		−27.17	
R = *o*-F	298	2.46	0.99956	4.50	−25.04	99.13
	307	2.58	0.99949		−25.93	
	316	2.72	0.99912		−26.83	

^a^ *R* is the correlation coefficient.

**Table 3:** Binding numbers of FDQL to HSA.

Compound	T (K)	*n*	*R* ^a^	*SD* ^b^
R = H	298	0.99314	0.99945	0.01088
	307	0.98423	0.99947	0.01059
	316	0.97492	0.99928	0.01220
R = *p*-F	298	0.97047	0.99914	0.01329
	307	0.98121	0.99942	0.01100
	316	0.98160	0.99947	0.01054
R = *m*-F	298	0.98976	0.99949	0.01043
	307	1.00621	0.99926	0.01276
	316	0.99960	0.99942	0.01126
R = *o*-F	298	0.94907	0.99959	0.00895
	307	0.97208	0.99936	0.01150
	316	0.98093	0.99884	0.01562

^a^ *R* is the correlation coefficient. ^b^ *SD* is the standard deviation for the *n* values.

**Table 4:** Effects of the site probe on the binding constants of FDQL to HSA.

Compound	Site Marker	*K*_a_ (×10^4^ M^−1^)	*R* ^a^	*SD* ^b^
R = H	Blank	2.16	0.99949	0.26651
	PB	1.88	0.99962	0.27020
	FA	1.04	0.99948	0.25962
	Dig	2.18	0.99938	0.24163
R = *p*-F	Blank	2.33	0.99945	0.23559
	PB	2.44	0.99960	0.23309
	FA	2.10	0.99976	0.22148
	Dig	2.43	0.99954	0.20943
R = *m*-F	Blank	2.73	0.99979	0.14653
	PB	2.08	0.99931	0.26689
	FA	1.65	0.99969	0.26159
	Dig	2.78	0.99955	0.18883
R = *o*-F	Blank	2.46	0.99956	0.18871
	PB	2.25	0.99922	0.25466
	FA	1.72	0.99972	0.22793
	Dig	2.54	0.99914	0.24946

^a^ *R* is the correlation coefficient. ^b^ *SD* is the standard deviation for the *K*_a_ values.

**Table 5:** The values of ΔΔ*H* and ΔΔ*S* in FDQL-HSA systems.

Compound	ΔΔ*H* (kJ·mol^−1^)	ΔΔ*S* (J·mol^−1^·K^−1^)
R = *p*-F	1.69	6.32
R = *m*-F	−1.65	−3.56
R = *o*-F	−2.64	−7.79

**Table 6:** Values of ΔΔ*G* at corresponding temperature in FDQL-HSA systems.

	T (K)	R = *p*-F	R = *m*-F	R = *o*-F
**ΔΔ*G* (kJ·mol^−1^)**	298	−0.20	−0.59	−0.32
	307	−0.25	−0.56	−0.25
	316	−0.30	−0.52	−0.18

**Table 7:** Conformation changes of the secondary structure of HSA with and without FDQL.

Sample	Secondary Structure (%)			
	α-Helix	β-Sheet	β-Turn	Random Coil
HSA	38.8	23.6	11.0	26.6
HSA + **3a** (1:2)	35.8	22.7	14.2	27.2
HSA + **3b** (1:2)	36.8	17.6	17.0	28.6
HSA + **3c** (1:2)	35.1	16.3	19.7	28.9
HSA + **3d** (1:2)	38.0	23.9	12.5	25.5

## Supplementary Materials

Supplementary materials can be accessed at: http://www.mdpi.com/1420-3049/21/10/1373/s1.
