# Supplementary material for: Comparative Studies of Interactions between Fluorodihydroquinazolin Derivatives and Human Serum Albumin with Fluorescence Spectroscopy
Source: Molecules. 2016 Oct 14;21(10):1373. doi: 10.3390/molecules21101373 (PMC6273767; doi:10.3390/molecules21101373)
Supplement: Supplementary file 1 [file molecules-21-01373-s001.pdf]

Supplementary Materials: Comparative Studies of Interactions between Fluorodihydroquinazolin Derivatives and Human Serum Albumin with Fluorescence Spectroscopy

Yi Wang, Meiqing Zhu, Feng Liu, Xiangwei Wu, Dandan Pan, Jia Liu, Shisuo Fan, Zhen Wang, Jun Tang, Risong Na, Qing X. Li, Rimao Hua and Shangzhong Liu

NMR Spectra of Intermediates and Target Compounds

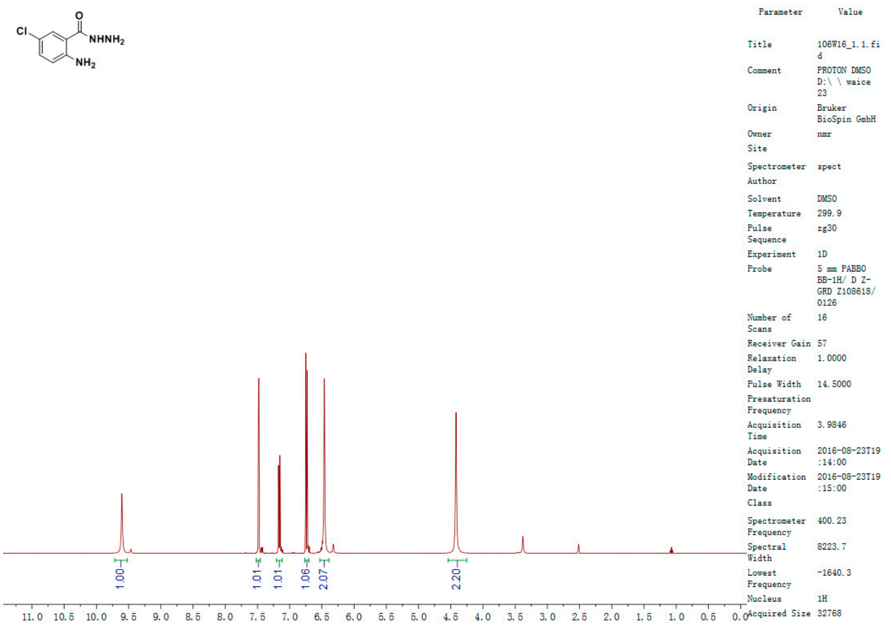

Figure S1. *2-Amino-5-chlorobenzohydrazide* (**1**) ^{1}H-NMR

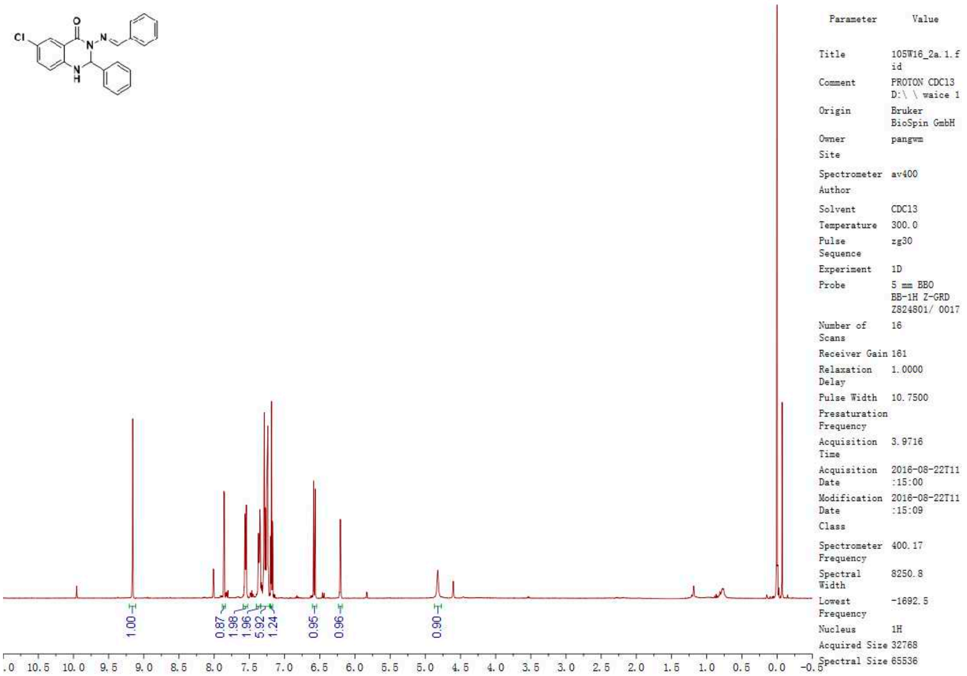

Figure S2. *3-(Benzylideneamino)-6-chloro-2-phenyl-2,3-dihydroquinazolin-4(1H)-one* (**2a**) ^{1}H-NMR.

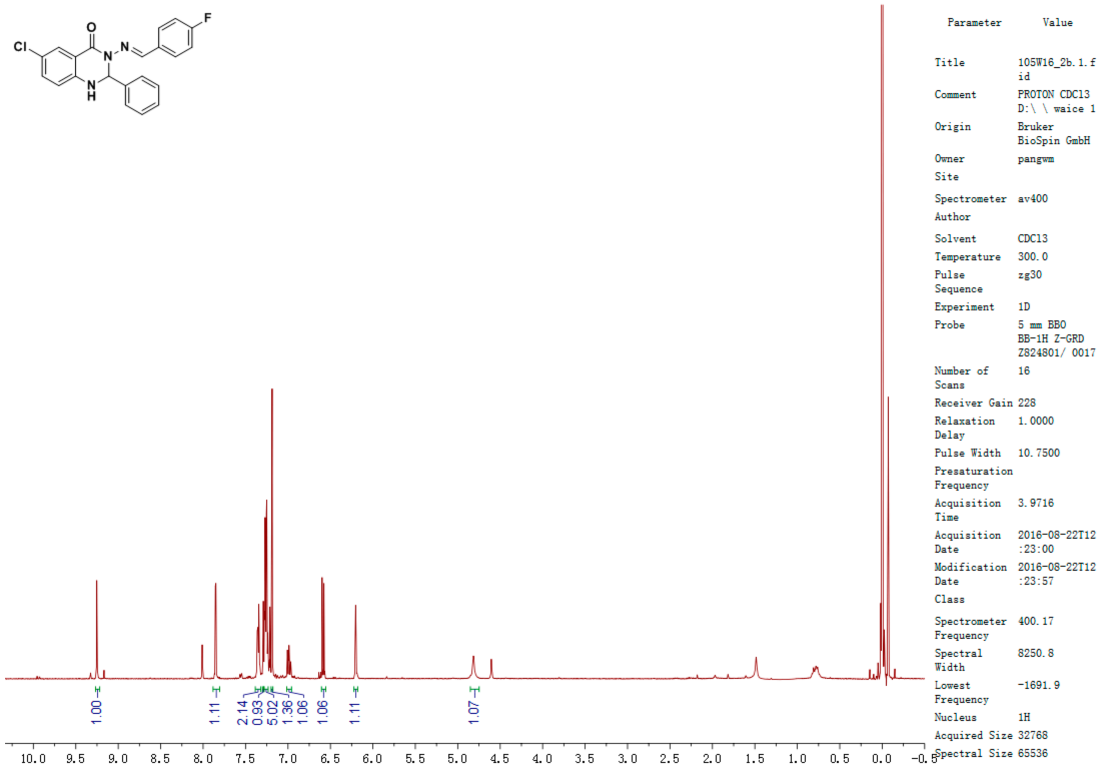

Figure S3. *6-Chloro-3-(2-fluorobenzylideneamino)-2-phenyl-2,3-dihydroquinazolin-4(1H)-one* (**2b**) ^{1}H-NMR.

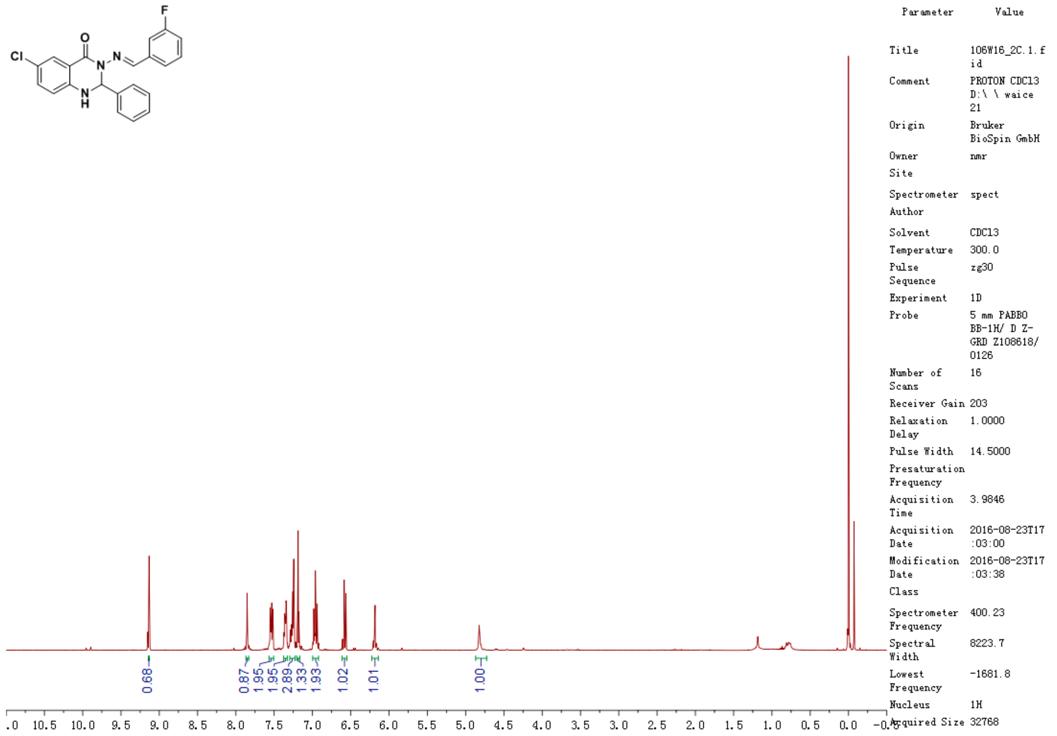

Figure S4. *6-Chloro-3-(3-fluorobenzylideneamino)-2-phenyl-2,3-dihydroquinazolin-4(1H)-one* (**2c**) ^{1}H-NMR.

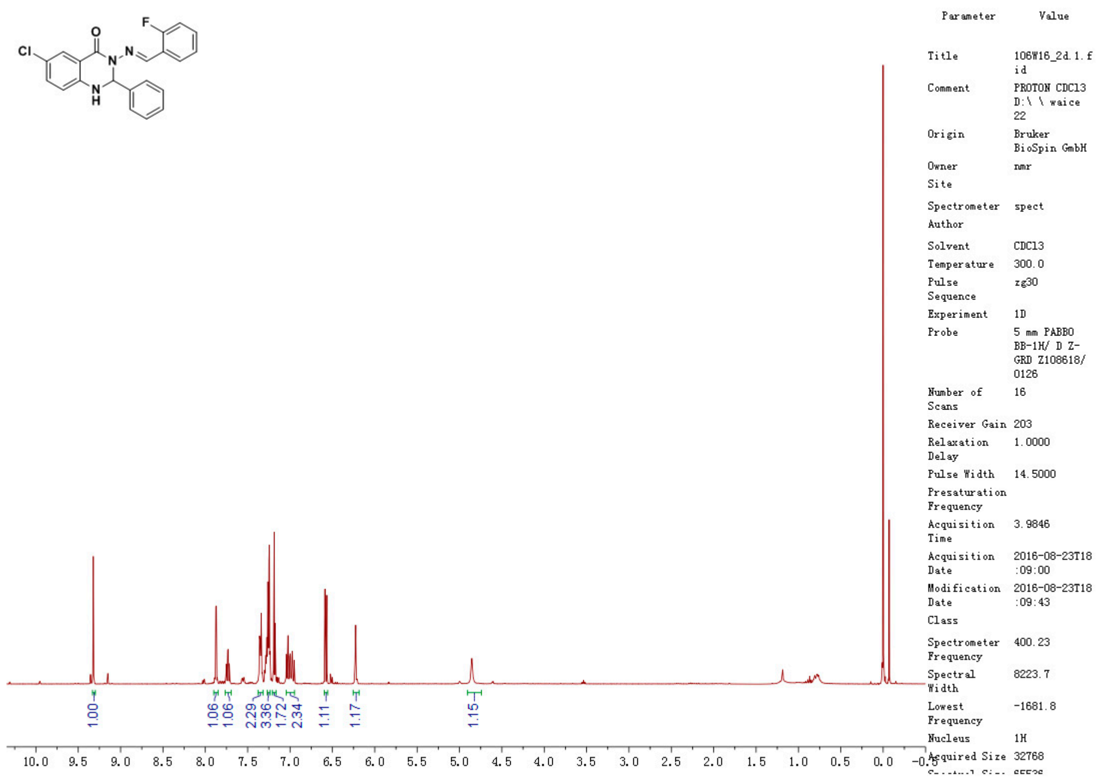

Figure S5. *6-Chloro-3-(4-fluorobenzylideneamino)-2-phenyl-2,3-dihydroquinazolin-4(1H)-one* (**2d**) ^{1}H-NMR.

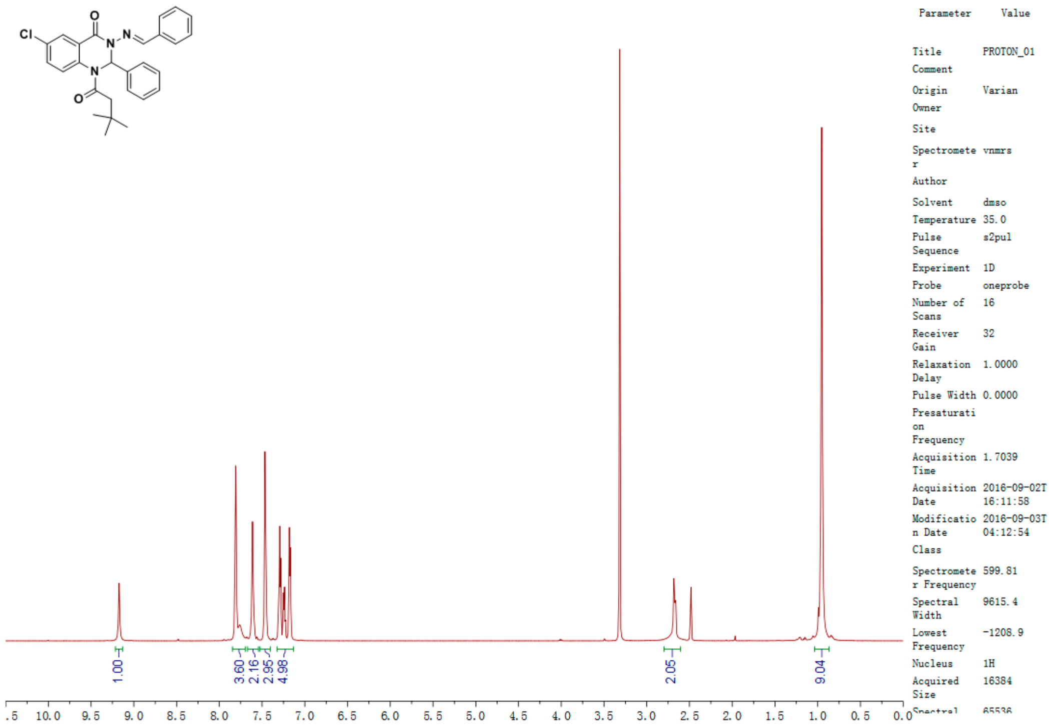

Figure S6. *3-(Benzylideneamino)-6-chloro-1-(3,3-dimethylbutanoyl)-2-phenyl-2,3-dihydroquinazolin-4(1H)-one* (**3a**) ^{1}H-NMR.

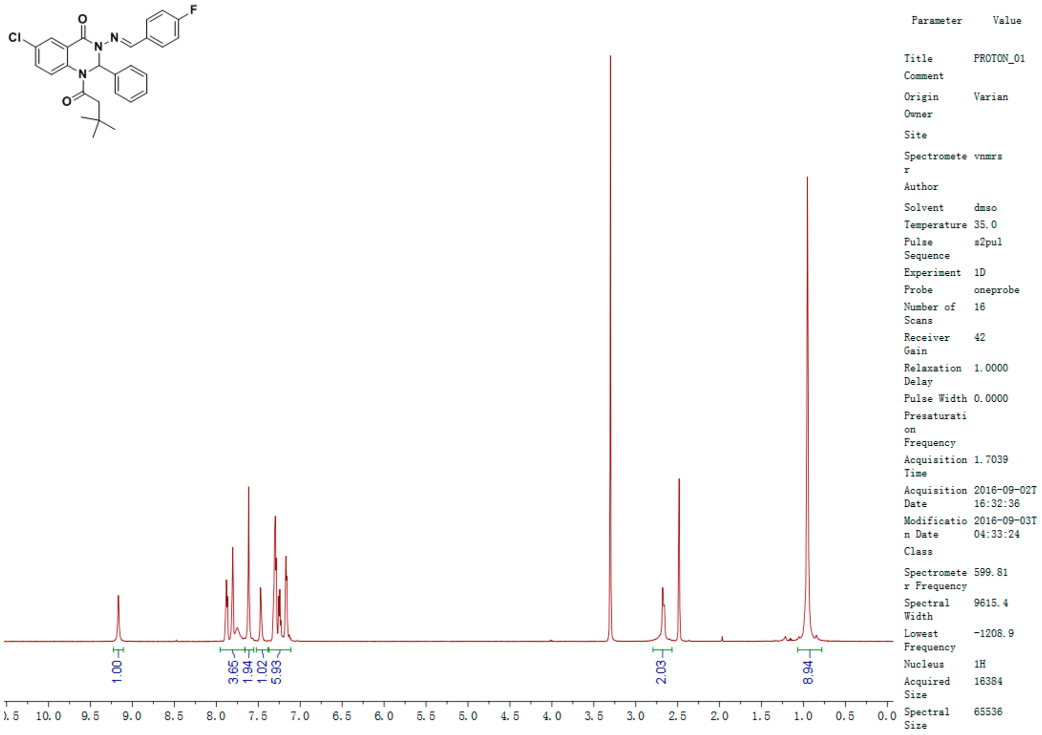

Figure S7. *6-Chloro-1-(3,3-dimethylbutanoyl)-3-(2-fluorobenzylideneamino)-2-phenyl-2,3-dihydroquinazolin-4(1H)-one* (**3b**) ^{1}H-NMR.

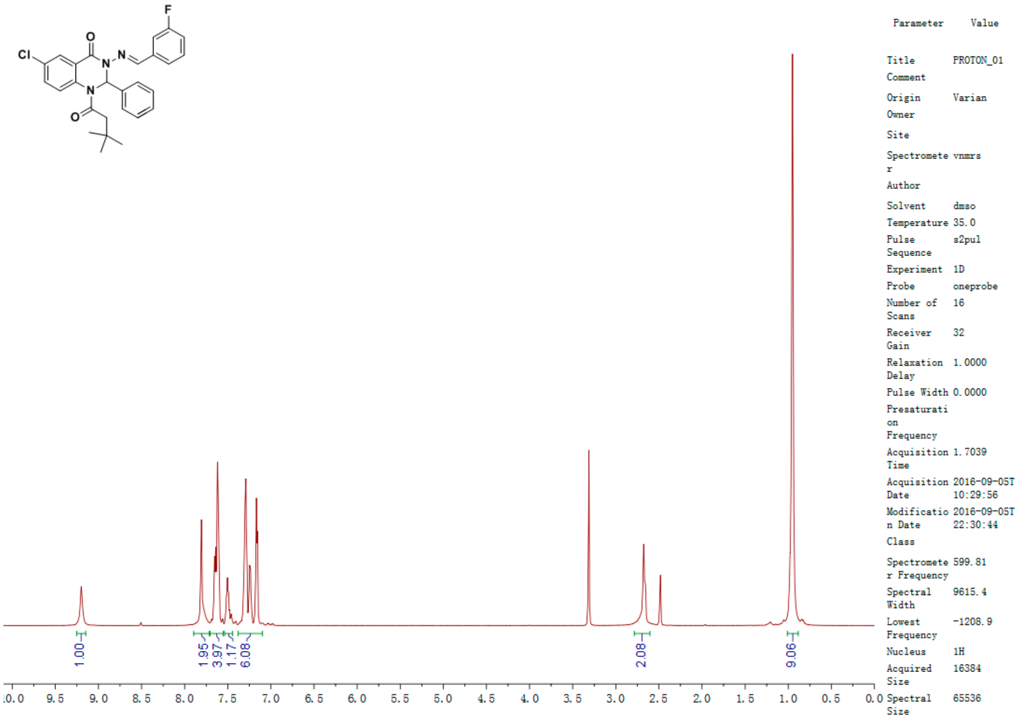

Figure S8. *6-Chloro-1-(3,3-dimethylbutanoyl)-3-(3-fluorobenzylideneamino)-2-phenyl-2,3-dihydroquinazolin-4(1H)-one* (**3c**) ^{1}H-NMR.

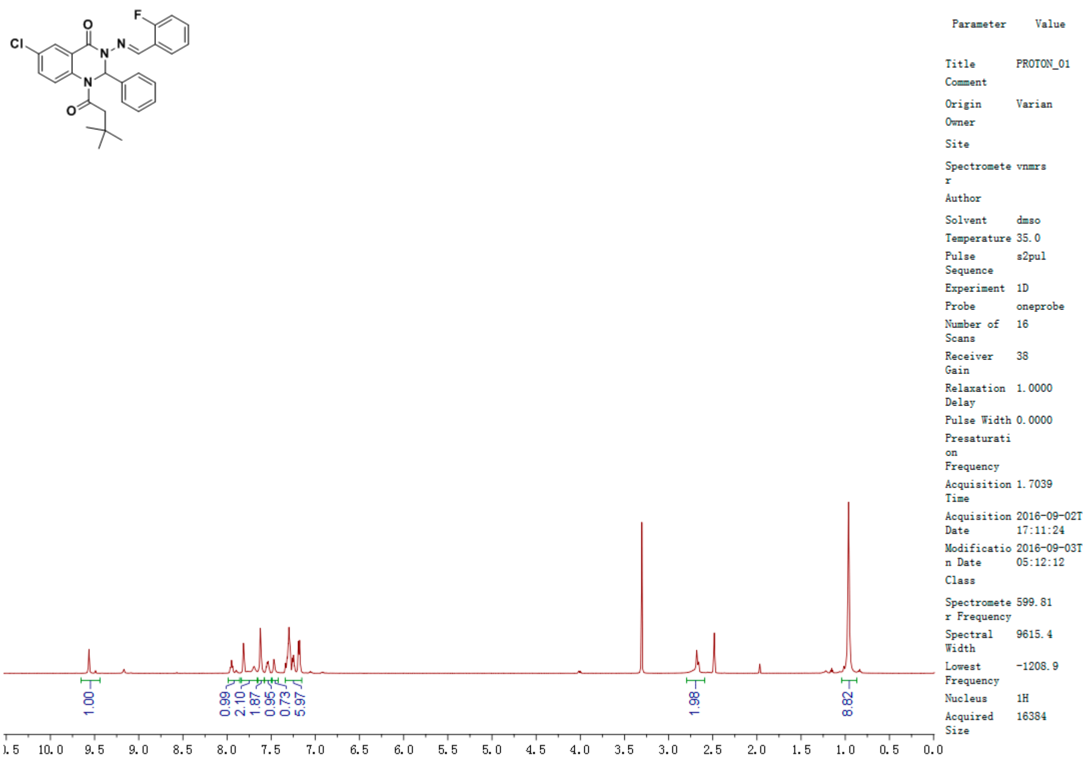

Figure S9. *6-Chloro-1-(3,3-dimethylbutanoyl)-3-(4-fluorobenzylideneamino)-2-phenyl-2,3-dihydroquinazolin-4(1H)-one* (**3d**) ^{1}H-NMR.

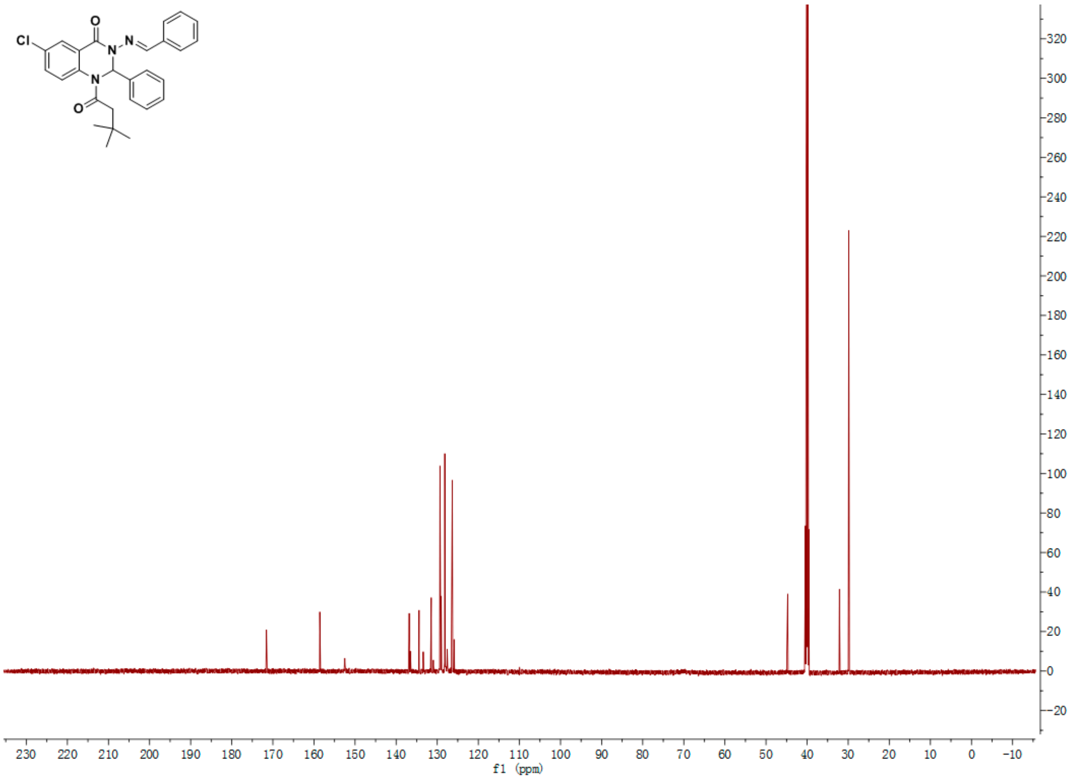

Figure S10. *3-(Benzylideneamino)-6-chloro-1-(3,3-dimethylbutanoyl)-2-phenyl-2,3-dihydroquinazolin-4(1H)-one* (**3a**) ^{13}C-NMR.

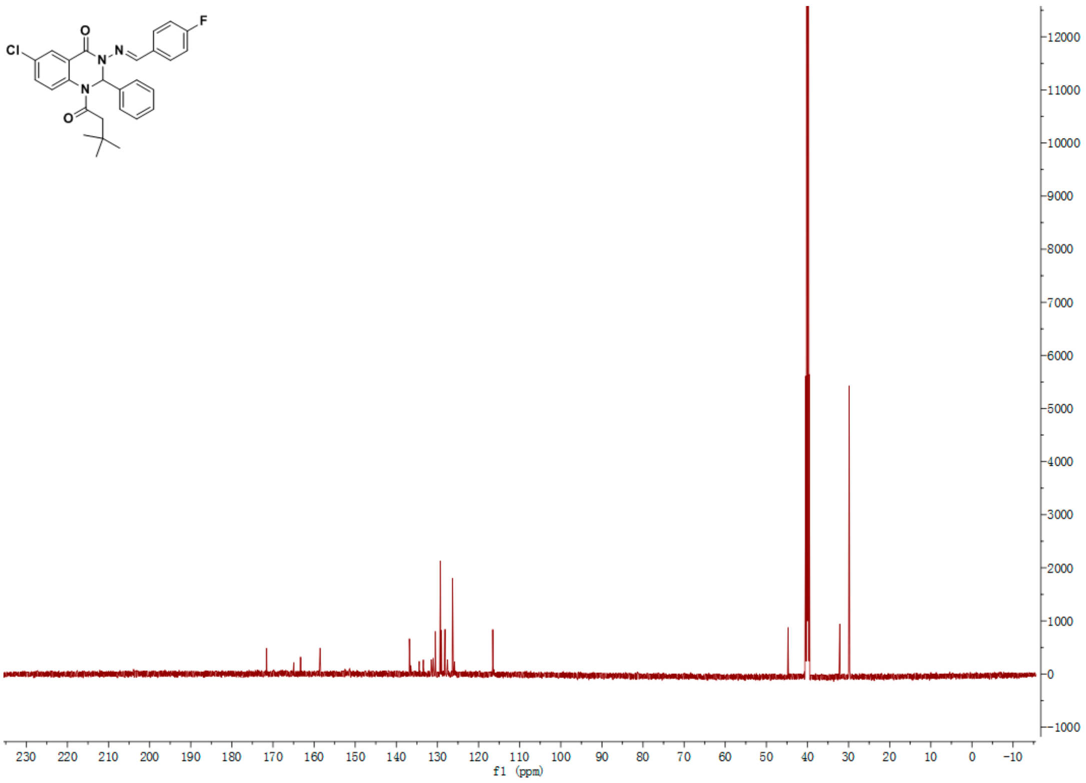

Figure S11. *6-Chloro-1-(3,3-dimethylbutanoyl)-3-(2-fluorobenzylideneamino)-2-phenyl-2,3-dihydroquinazolin-4(1H)-one* (**3b**) ^{13}C-NMR.

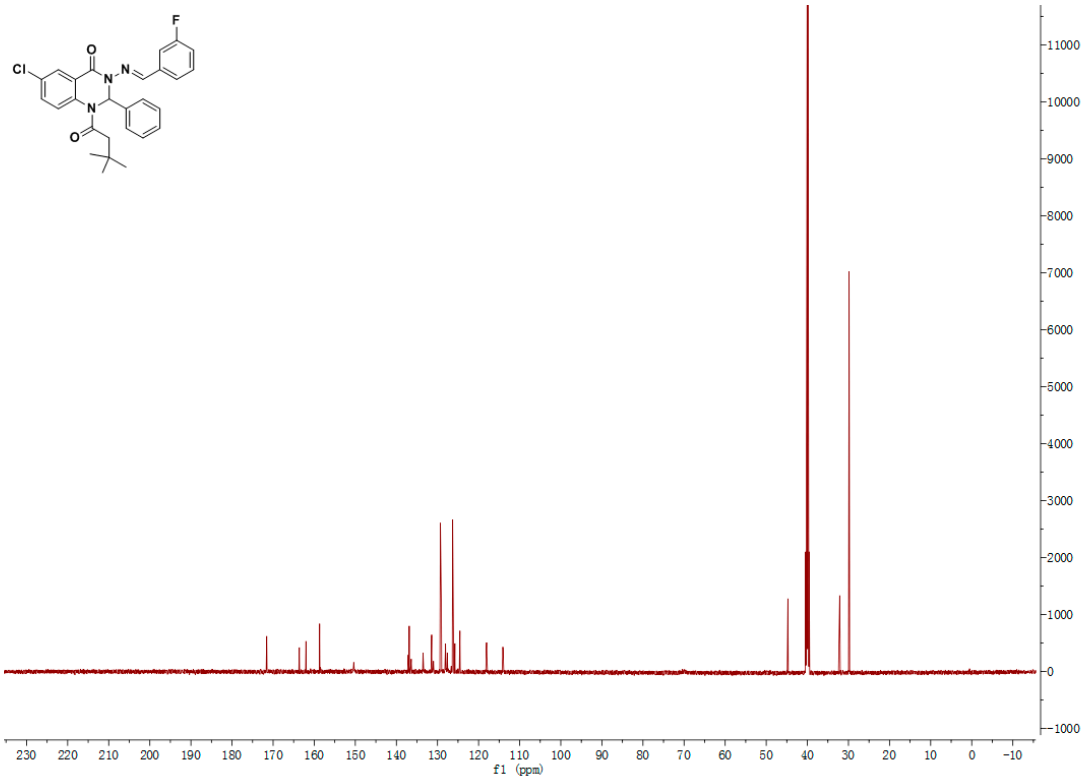

Figure S12. *6-Chloro-1-(3,3-dimethylbutanoyl)-3-(3-fluorobenzylideneamino)-2-phenyl-2,3-dihydroquinazolin-4(1H)-one* (**3c**) ^{13}C-NMR.

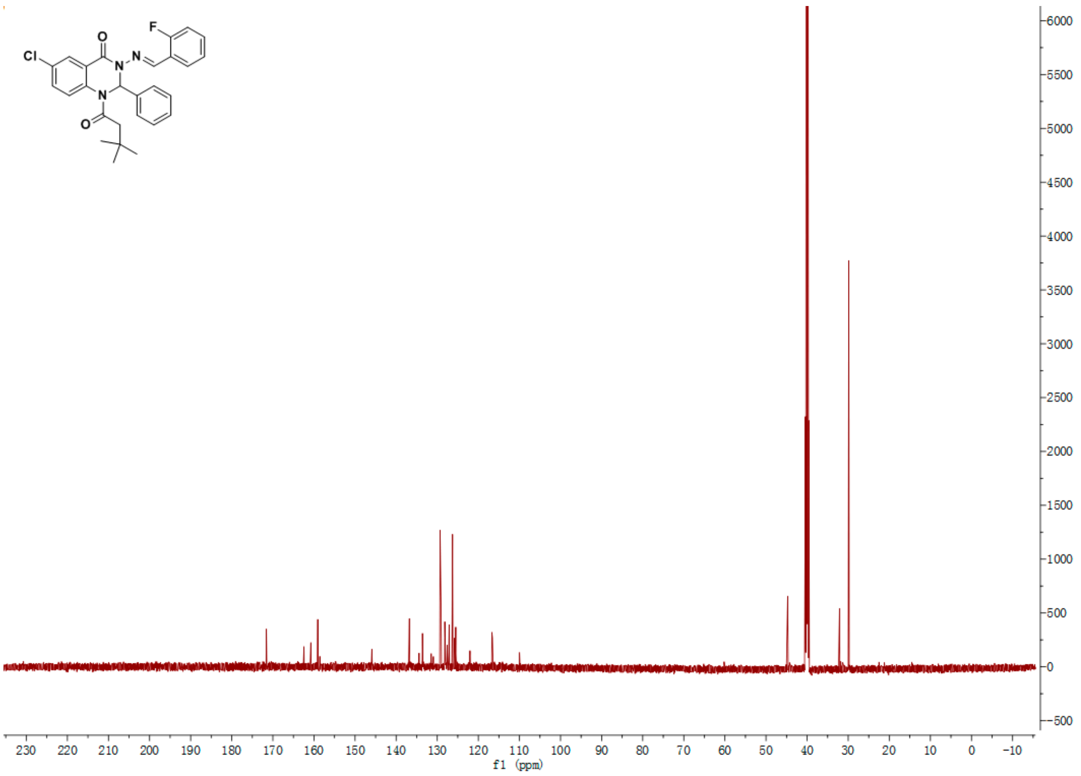

Figure S13. *6-Chloro-1-(3,3-dimethylbutanoyl)-3-(4-fluorobenzylideneamino)-2-phenyl-2,3-dihydroquinazolin-4(1H)-one* (**3d**) ^{13}C-NMR.
